# Management of Childhood Thyroid Nodules: Surgical and Endocrinological Findings in a Large Group of Cases

**DOI:** 10.4274/jcrpe.4272

**Published:** 2017-09-01

**Authors:** Emre Divarcı, Ülgen Çeltik, Zafer Dökümcü, Orkan Ergün, Geylani Özok, Samim Özen, Damla Gökşen Şimşek, Şükran Darcan, Nazan Çetingül, Aylin Oral, Yeşim Ertan, Bengü Demirağ, Ahmet Çelik

**Affiliations:** 1 Ege University Faculty of Medicine, Department of Pediatric Surgery, İzmir, Turkey; 2 Ege University Faculty of Medicine, Division of Pediatric Endocrinology, İzmir, Turkey; 3 Ege University Faculty of Medicine, Division of Pediatric Oncology, İzmir, Turkey; 4 Ege University Faculty of Medicine, Department of Nuclear Medicine, İzmir, Turkey; 5 Ege University Faculty of Medicine, Department of Pathology, İzmir, Turkey; 6 Dr. Behçet Uz Children’s Hospital, Division of Pediatric Oncology, İzmir, Turkey

**Keywords:** Thyroid nodule, thyroidectomy, papillary carcinoma, frozen section, fine-needle aspiration biopsy

## Abstract

**Objective:**

The management of childhood thyroid nodules is still a big challenge for clinicians. In this study, we aimed to present our surgical and endocrinological experience in more than one hundred pediatric cases.

**Methods:**

A retrospective analysis of patients admitted with a thyroid nodule between 2006 and 2014 was performed. Detailed ultrasonography and fine-needle aspiration biopsy (FNAB) were the cornerstones of the diagnostic approach.

**Results:**

One hundred-three children (72 female, 31 male) with a mean age of 13.1±3.6 years (3-18 years) were admitted to our center. Management strategy was surgery in 58 patients and follow-up in 45 patients. Mean nodule size was 17±12.7 mm (2-45 mm). The diagnoses were listed as benign solitary nodule (48 patients), thyroid carcinoma (26 patients), multinodular goiter (23 patients), Hashimoto thyroiditis (4 patients), and Graves’ disease (2 patients). Surgical procedures were nodulectomy/lobectomy (32 patients), total thyroidectomy (TT) (13 patients), or TT+ neck dissection (13 patients). The rate of malignancy was 25% in the total group and 44% in the surgery group. The malignancy rate was higher in patients younger than 12 years compared to older children (41% vs. 17%, p=0.040). Metastasis was seen in 38% of the malignant nodules. Postoperative complications were transient hypocalcemia (8%), permanent hypocalcemia (1.7%), and unilateral vocal cord paralysis (1.7%). Recurrence or mortality was not encountered in the 5.4±1.2-year follow-up period.

**Conclusion:**

Thyroid nodule in a child requires an aggressive diagnostic approach due to increased risk of malignancy and metastasis. Intraoperative frozen section examination must be done as a useful adjunct to determine the surgical strategy. Incidence of complications is small in thyroid surgery when performed by experienced surgeons.

**What is already known on this topic?**

The management of thyroid nodules in children is still a big challenge for clinicians. The rarity of this clinical entity limits the surgical and endocrinological experience in most pediatric centers.

**What this study adds?**

In this study, we aimed to present our surgical and endocrinological experience in more than one hundred pediatric cases. We emphasized the importance of a standard management approach including detailed ultrasonography, fine-needle aspiration biopsy, and frozen section examination due to high rates of malignancy and metastasis in children.

## INTRODUCTION

The management of thyroid nodules in children is still a big challenge for clinicians. The treatment strategies were usually derived from adult guidelines until the recent publication of pediatric guidelines for the management of thyroid nodules in children by the American Thyroid Association (ATA) (1,2,3). While most of the main principles in the treatment of pediatric cases are similar to those of adults, there are certain specific and distinctive characteristics in the diagnosis and surgical treatment of children with thyroid nodules.

Thyroid nodules are seen less frequently in childhood than adults (0.2-5% of children, 13% of adolescents) (4). However, the risk of malignancy of a pediatric thyroid nodule is higher than that in an adult patient (7-15 % in adults vs. 22-26% in children) (1,5,6). The rarity of this clinical entity limits the acquisition of surgical and endocrinological experience in most pediatric centers (7,8,9). In this study, we aimed to present our surgical and endocrinological experience in a large group of pediatric cases with thyroid nodules.

## METHODS

A retrospective analysis of patients with a thyroid nodule who presented or referred to our center between 2006 and 2014 was performed. The medical records of patients including preoperative, intraoperative, and postoperative data were reviewed. We analyzed the following information: preoperative data which comprised demographics, clinical symptoms, physical examination, family history, radiological features, fine-needle aspiration biopsy (FNAB) results; intraoperative data which included frozen section findings and surgical procedures; and postoperative data which covered histopathological findings, complications, follow-up findings, and final prognosis.

The management of a thyroid nodule initially started with identification of the malignancy risk for differentiated thyroid carcinoma (DTC). Preoperative work-up consisted of clinical and family history, physical examination, detailed thyroid and neck ultrasonography, laboratory tests (thyroid hormones, thyroid antibodies, calcitonin, and electrolyte levels), and FNAB.

**Figure 1:**
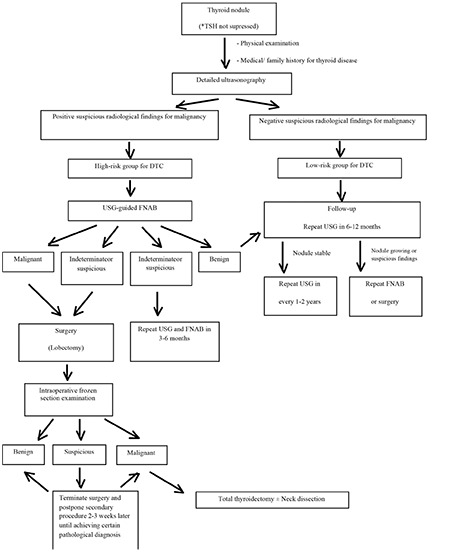
Management protocol in a pediatric patient with a thyroid nodule DTC: differentiated thyroid carcinoma, USG: ultrasonography, FNAB: fine-needle aspiration biopsy, TSH: thyroid-stimulating hormone

Thyroid nodules were identified as low risk or high risk for DTC by the preoperative diagnostic studies listed above. Ultrasonography findings including increased nodular size, hypoechogenicity, invasive and irregular nodule margins, increased nodular blood flow, microcalcifications, and abnormal cervical lymph nodes were accepted as potential signs of high risk malignancy. Patients with such findings were investigated by ultrasonography-guided FNAB and/or surgical sampling. Patients with thyroid nodules underwent surgery or follow-up in accordance with this management protocol (Figure 1).

Patients in the follow-up group with low risk for DTC were followed by ultrasonography and laboratory tests for 6-12-month periods. Patients with high-risk thyroid nodules underwent surgical excision by local nodule excision/lobectomy. In the earlier period of the study, we preferred local nodule excision, but subsequently we began to perform lobectomy as a standard procedure. We performed intraoperative frozen section examination in all of the nodules to identify the histopathological diagnosis during surgery. Patients with malignant results underwent total thyroidectomy and those with metastatic cervical lymph nodes underwent central or lateral neck dissection. In benign results on frozen section examination, lobectomy was accepted to be sufficient to terminate the surgery. In suspicious results on frozen biopsy, surgery was terminated and a second surgical procedure was planned for 2-3 weeks later to ascertain the pathological diagnosis. Radioactive iodine therapy was applied in the postoperative period to all patients with thyroid malignancy. The dose of the radioiodine therapy was determined by body weight (37-74 MBq/kg).

All parents and adolescent patients gave their informed consent prior to their inclusion in the study. SPSS for Windows 20.0 was used for the statistical analysis. Pearson’s chi-square test was also used in the data analysis. A p-value lower than 0.05 was considered to be statistically significant.

## RESULTS

During the time period between 2006 and 2014, a total of 103 children (72 female, 31 male) were admitted to our center with a thyroid nodule. The diagnostic management protocol presented above was used to investigate the patients and to identify the potential risk for DTC. Fifty-eight patients with high risk for DTC underwent surgery and were labelled as the surgery group. Forty-five patients with low risk for DTC were followed without surgical intervention and named as the follow-up group. The data pertaining to the preoperative, intraoperative, and postoperative periods are presented below.

### Preoperative Data

Mean age of all patients was 13.1±3.6 years (3-18 years) (Table 1). Mean ages for the surgery and follow-up groups were 12.6±3.8 years (3-18 years) and 13.8±3.2 years (4-18 years), respectively (p=0.086). There was a female dominancy (2-3/1 ratio) in the total group. Visible or palpable swelling in the neck was the presenting admission symptom in 70 patients (68%). Twenty-three patients were diagnosed during examination for an increased risk for thyroid pathologies due to a positive medical or family history (21%). Ten patients had atypical symptoms like growth retardation or obesity and were identified incidentally (11%).

Twenty-four patients had a family history for thyroid diseases (23%). Twenty of these patients had multinodular goiter and four patients had a positive family history for DTC (2 with medullary carcinoma and 2 with papillary carcinoma). The patient’s medical history or his/her family history were not found to be an independent risk factor for malignancy of a thyroid nodule (p=0.931). Seven patients had a suspicious individual medical history for thyroid disorders. Four of them had been treated for a previous Hodgkin lymphoma or neuroblastoma and had a history of radiotherapy to the neck 3 to 12 years ago. The size of the nodules in these patients who had a history of radiotherapy was smaller than 1 cm (6-8 mm). However, all of the nodules were malignant. The other three patients had underlying thyroid disorders as Hashimoto thyroiditis (2 patients) or Graves’ disease (1 patient).

Thyroid ultrasonography was performed in all patients in the preoperative work-up. Mean nodule size was 17±12.7 mm (2-45 mm) for the total group. In the group who underwent surgery, mean nodule size was 22.1±12.2 mm (5-45 mm) and larger than that of the follow-up group, which was 10.2±10.1 mm (2-30 mm). Mean nodule size was significantly larger in the surgery group (p=0.000). Chest CT scan was applied to five patients due to the high risk for distant metastasis. Two of them had pulmonary metastasis.

Thyroid scintigraphy was performed in 40 patients. Eighteen patients had hypoactive cold nodules. DTC was detected in only four of these 18 patients (22%). Fourteen patients had hyperactive hot nodules on scintigraphy. Seven of 14 patients had DTC (50%). The results of our patients were not in accordance with classical knowledge about thyroid scintigraphy.

In this group of patients, FNAB was performed more frequently in recent years. Thirty-one patients underwent FNAB. The results of FNAB revealed that the histology was benign (16 patients), non-diagnostic (10 patients), suspicious (4 patients), and malignant (1 patient). Patients with malignant results or suspicious results in biopsy but who showed characteristic ultrasonographic features for malignancy underwent surgical excision.

Thyroid hormone levels were normal in most of the patients -98 of 103 patients were euthyroid (95%). Two patients with benign nodules had a mild increase in thyroid-stimulating hormone (TSH) levels. One patient with Hashimoto thyroiditis had a significantly increased TSH level (>100 µIU/L). TSH levels were low in two patients with multinodular goiter.

### Intraoperative Data

The initial surgical procedure in a group of patients was local excision of the suspected lesion by nodulectomy or lobectomy. This surgical sampling by local nodule excision was performed in the early period of the study (15 patients, 25%), but we preferred lobectomy primarily in recent years (43 patients, 75%) (Table 2). Frozen section examination was performed in all patients who underwent surgery. The results of the frozen examination during surgery showed that the nodule was benign in 28 patients, malignant in 23 patients, and suspicious in seven patients. Surgery was terminated in the patients with benign and/or suspicious results. Seven patients with intraoperative suspicious results underwent further histopathological exploration in the postoperative period. Four of these 7 patients had benign results with a diagnosis of cystic nodular goiter (3 patients) or follicular adenoma (1 patient) and did not require any additional surgical intervention. However, three patients with suspicious results on frozen examination had subsequent malignant results as papillary carcinoma (2 patients) and follicular carcinoma (1 patient) which necessitated total thyroidectomy 2-3 weeks after the initial surgery.

Thus, 26 of the patients had malignancy and required total thyroidectomy. Mean age in these patients with malignant nodules was statistically lower than that of the patients with benign nodules (11.9±3.6 vs. 13.6±3.5) (p=0.047). Most of these patients were female (20 patients, 77%). Median nodule size was similar in patients with benign and malign nodules as 14 mm (2-45 mm and 5-50 mm).

The overall rate of malignancy in this group of patients with thyroid nodules was 25% (26/103). The rate of malignancy in the surgery group was 44.8% (26/58). The standard surgical procedure was total thyroidectomy in patients without cervical lymph node metastasis. In patients with preoperative suspicion of metastatic lymph nodes on ultrasonography, frozen section examination was performed to lymph nodes. Also, patients with suspected nodules during surgery were investigated for metastasis. 13 patients underwent neck dissection for suspicion of lymph node metastasis (22%). The surgical approach was central dissection in 10 patients and additional lateral neck dissection in 3 patients.

### Postoperative Data

The spectrum of diagnosis ranged from solitary benign nodules, multinodular goiter, medical disorders like Graves’ disease and Hashimoto thyroiditis to malignancies such as papillary, medullary, and follicular carcinoma (Table 3). Most patients had benign etiologies (75%, 77/103). The most common disorder was a solitary benign thyroid nodule in 48 patients (46%). Twenty-eight of these patients were followed with ultrasonography and FNAB. The other 20 patients with benign solitary nodules underwent surgical excision. The diagnosis was multinodular goiter in 23 of these patients. Follow-up (15 patients) or surgery (8 patients) were the preferred management approaches in multinodular goiter. Surgery was preferred in cases with a large goiter located bilaterally. Total thyroidectomy was preferred in these 8 patients for cosmetic concerns. A decision for surgery (2 patients) or follow-up (2 patients) was made in the 4 patients with Hashimoto thyroiditis according to the medical condition of the patients. Two children with Graves’ disease underwent subtotal thyroidectomy. Twenty-six patients had malignant etiologies as papillary carcinoma (22 patients), medullary carcinoma (2 patients), and follicular carcinoma (2 patients). Metastasis was detected in 10 of these 26 patients during initial diagnosis (38%). Cervical lymph nodes were involved in all of these 10 patients. In addition, 2 of these patients had diffuse lung metastasis and underwent radioactive iodine therapy. Patients with medullary carcinoma had a positive family history for medullary carcinoma. These patients did not have any clinical symptoms or physical examination findings, but 5 and 10 mm solitary thyroid nodules were detected on ultrasonography. Prophylactic thyroidectomy was preferred in these patients, and medullary carcinoma was identified on pathological examination. Follicular carcinoma was detected in 2 patients. The size of the nodules were larger as 37 and 45 mm in these 2 patients. FNAB was performed in one of them revealing suspicious cytology. No surgical complications occurred in patients with follicular and medullary carcinomas. All of the complications were seen in patients with papillary carcinomas.

The age of the children with a thyroid nodule varied from 3 years to 18 years in this in this group of patients. The malignancy rate was 41 % (14/34) in patients of ages ≤12 years and higher than the rate of 17% (12/69) in patients ≥12 years. This difference was statistically significant (p=0.040).

In the early postoperative period, all patients were monitored for possible complications such as hypocalcemia and vocal cord paralysis. Permanent unilateral vocal cord paralysis was detected in one patient (1.7%). The hoarseness did not require tracheostomy. Transient hypocalcemia was determined in five patients (8%) and resolved spontaneously in the postoperative period. Permanent hypocalcemia occurred in one patient and required permanent oral calcium and calcitriol replacement (1.7%).

Mean postoperative follow-up was 5.4±1.2 years (2-10 years). No complications were noted in the late postoperative period. Two patients who had undergone nodule excision with the diagnosis of multinodular goiter required total thyroidectomy in the late postoperative period due to cosmetic concerns. Recurrence or mortality was not encountered in any of the patients (0%).

## DISCUSSION

The management protocol for a thyroid nodule in children starts with a detailed preoperative examination which usually necessitates an operative procedure and continues with long-term follow-up in the postoperative period. The malignancy rate and metastasis risk for a thyroid nodule in a child is higher than those in an adult (1,5,6). In our study, the rates of malignancy and metastasis were identified as 25% and 44 %, respectively. Lower age is a significant risk factor for malignancy in a thyroid nodule. In this study, we found that there was a two times increased risk in children younger than 12 years old as compared to those who were older (41% vs. 17%). Therefore, a more aggressive approach is necessary in younger children. The metastasis rate was also higher. For these reasons, the nature of a thyroid nodule needs to be detected immediately to achieve effective treatment. A history of neck radiotherapy is a significantly important risk factor for malignancy in a thyroid nodule. In our study, papillary carcinoma was identified in all of the nodules which were smaller than 1 cm in patients with a history of a previous neck radiotherapy.

A management protocol should be initiated with identification of the risk of malignancy in a nodule. Detailed thyroid ultrasonography is essential to reveal the risk by demonstration of specific radiological findings like microcalcifications, invasive nodule borders, hypoechogenicity, and increased vascularity (10). Also, the nodule size must be monitored regularly to detect any enlargement as a critical parameter for additional diagnostic intervention. FNAB should be performed under ultrasonography guidance (11,12,13,14). After identification of high risk for DTC, surgical sampling by lobectomy must be preferred as an initial surgical intervention. Nodulectomy is given up in solitary nodules to avoid the surgical risks of subsequent surgical procedures with a new nodule formation in residual thyroid lobe. Routine intraoperative frozen section examination must be applied in all patients with an increased risk of malignancy in a thyroid nodule (15,16,17). In our study, we achieved a high rate of useful results by frozen examination which were helpful in deciding on the surgical strategy during surgery. Surgery is terminated in cases with benign results. Suspicious results which cannot identify the risk of malignancy necessitate further pathological examination and possibly indicate a need for complete total thyroidectomy.

Frozen section examination during surgery is a useful adjunct in the decision of surgical strategy but has some limitations (18). Lobectomy must be performed to investigate the nodule with extranodular margins. Frozen section biopsy could diagnose classical papillary thyroid carcinoma (PTC) successfully. However, it has limitations for the diagnosis of follicular variant PTC and may not identify follicular thyroid carcinoma which requires the evaluation of the entire lesion and vascular/capsular invasion (19,20).

In the early period of our study, we performed routine thyroid scintigraphy as a diagnostic tool. We found conflicting results about the activity of nodules as cold or hot. Routine scintigraphy is not essential in the management of thyroid nodules. In the ATA pediatric guideline, routine scintigraphy is not recommended in patients with normal TSH levels (3).

The complication rate in thyroid surgery is very low in experienced hands. The most frequent complication we encountered was transient hypocalcemia in five patients in the early postoperative period (8%) which resolved spontaneously. Unilateral vocal cord paralysis occurred in one patient with an incidence of 1.7%. This patient’s diagnosis was follicular variant papillary carcinoma and she had bilateral fixed large nodules. Permanent hypocalcemia was seen in one patient with the diagnosis of solid variant papillary carcinoma. In conformity with previous reports from experienced teams, no major complications such as bleeding, signs indicating a need for tracheostomy or wound infection during or after surgery developed in any of our patients (21).

Thus, we conclude that children admitted with a thyroid nodule must be evaluated by an experienced multidisciplinary team including experts in pediatric endocrinology, radiology, pathology, oncology, nuclear medicine, and surgery. A thyroid nodule in a child requires an aggressive diagnostic approach due to increased risk of malignancy and metastasis. The cornerstone of the diagnostic approach is a detailed thyroid and neck ultrasonography. Enlarged nodules larger than 1 cm and nodules which have specific ultrasonographic findings need to undergo ultrasonography guided FNAB. Also, risk of malignancy is higher in patients with a previous history of radiotherapy to the neck and in those with nodules smaller than 1 cm. In patients with a high-risk thyroid nodule, surgical intervention must be performed under frozen section examination guidance during surgery. Malignant results on frozen examination necessitate total thyroidectomy. Also, metastatic cervical lymph nodes require central or lateral neck dissection.

## Figures and Tables

**Table 1:**
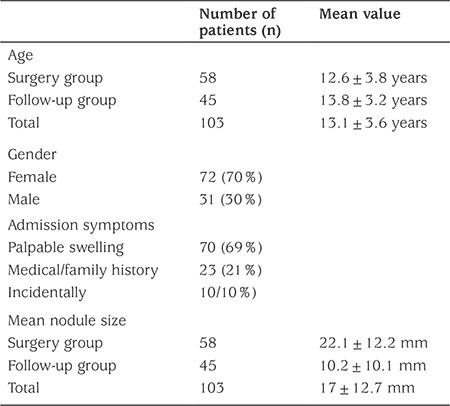
Preoperative data

**Table 2:**
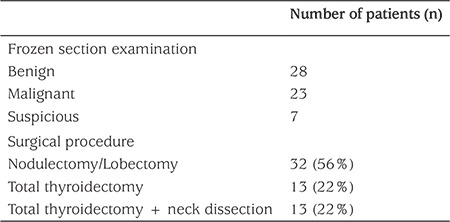
Intraoperative data

**Table 3:**
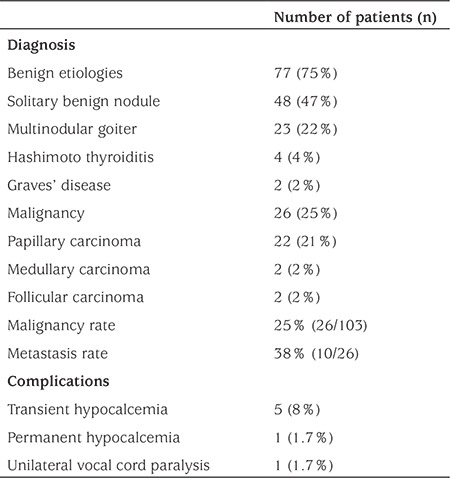
Postoperative data
